# High-Velocity, Accentuated Eccentric, or Maximal Elastic Band Resistance Training? Effects of Resistance Training Modalities on Bone Health, Isokinetic Strength, and Systemic Biomarkers in Sedentary Older Adults: A Comparative Study

**DOI:** 10.3390/healthcare13233129

**Published:** 2025-12-01

**Authors:** Angel Saez-Berlanga, Javier Gene-Morales, Ana María Teixeira, Ruth Jiménez-Castuera, Andrés Gené-Sampedro, Alvaro Juesas, Pedro Gargallo, Oscar Caballero, Julio Fernandez-Garrido, Carlos Alix-Fages, Pablo Jiménez-Martínez, Juan C. Colado

**Affiliations:** 1 Department of Physical Education and Sports, Universitat de València, 46010 Valencia, Spain; angel.saez@uv.es (A.S.-B.); juan.colado@uv.es (J.C.C.); 2 Research Group in Prevention and Health in Exercise and Sport (PHES), Universitat de València, 46010 Valencia, Spain; alvaro.juesastorres@uchceu.es (A.J.); pedro.gargallo@uv.es (P.G.); c.alix@icen.es (C.A.-F.); p.jimenez@icen.es (P.J.-M.); 3 CIPER -Interdisciplinary Center for the Study of Human Performance, Faculty of Sport Sciences and Physical Education, University of Coimbra, 3040-256 Coimbra, Portugal; 4 Faculty of Sport Sciences, University of Extremadura, 10003 Cáceres, Spain; ruthji@unex.es; 5 Department of Optics, Optometry and Vision Sciences, University of Valencia, 46100 Valencia, Spain; andres.gene@uv.es; 6 Research Institute on Traffic and Road Safety, University of Valencia, 46022 Valencia, Spain; 7 Department of Education Sciences, CEU Cardenal Herrera University, 46115 Castellón, Spain; 8 Nursing Department, Faculty of Nursing and Podiatry, University of Valencia, 46010 Valencia, Spain; oscar.caballero@uv.es (O.C.); julio.fernandez@uv.es (J.F.-G.); 9 Department of Health Research, ICEN Research Center, 38002 Santa Cruz de Tenerife, Spain

**Keywords:** bone turnover markers, inflammaging, neuroplasticity, elastic band resistance training, rating of perceived exertion, older adults

## Abstract

**Objectives**: To examine three elastic band resistance training (EB-RT) modalities—high-velocity (HVRT), accentuated eccentric (Aecc), and maximal strength (Max)—on bone health, strength, redox-inflammatory profile, and neuroplasticity in sedentary older adults. **Methods**: Sixty-one participants (69.41 ± 4.61 years) were randomly assigned to HVRT (*n* = 21), Aecc (*n* = 13), Max (*n* = 10), or passive controls (*n =* 17). Training was conducted three times a week for 16 weeks. Sessions included four sets of alternating upper- and lower-limb EB exercises, with intensity guided by the OMNI–RES EB scale. HVRT emphasized explosive concentric actions [~70% one-repetition maximum (1RM); 3–4 rating of perceived exertion in the first repetition (RPE-1)]. Aecc performed 5 s eccentric overload [>100% 1RM; 7–8 RPE-1]. Max employed controlled 2 s concentric/eccentric actions [~80–85% 1RM; 7–8 RPE-1]. **Results**: All training groups improved isokinetic strength (*p* < 0.01, *g =* 0.91–2.40). HVRT increased brain-derived neurotrophic factor (BDNF) (*p* = 0.019, *g* = 0.42) and glutathione peroxidase (GPx) (*p* < 0.001, *g* = 0.31). Aecc elicited the strongest osteoanabolic and antioxidant effects (P1NP, *p =* 0.001, *g* = 1.21; β-CTX, *p <* 0.001, *g* = 1.82; F2-isoprostanes, *p =* 0.007, *g* = 0.94). Max induced moderate bone turnover benefits (P1NP, *p =* 0.005, *g* = 1.08; β-CTX, *p <* 0.001, *g* = 1.12), but no GPx or BDNF gains. Controls maintained or declined all variables. **Conclusions**: EB-RT over 16 weeks improved most outcomes overall, showing modality-specific trends: HVRT favored neuroplasticity, Aecc enhanced redox-inflammatory and bone remodeling responses, and Max improved strength and bone health. These findings support elastic band resistance training as a safe and individualized strategy for healthy aging.

## 1. Introduction

The concurrent deterioration of muscle and bone forms an osteomuscular continuum associated with falls, fractures, and disability in older adults [1]. Resistance training (RT) serves as a mechanosensitive stimulus that modulates both neuromuscular performance and bone remodeling [2]. Unlike aerobic training, RT enhances maximal strength and promotes osteogenesis via strain-driven osteocytic mechanotransduction [2]. Framed within the osteomuscular continuum associated with falls, fractures, and disability in aging, the study addresses a public health priority by evaluating elastic band resistance training (EB-RT) modalities that are feasible, low-cost, and demonstrably safe for this population [3].

Functional capacity in older adults depends largely on muscular power and maximal strength, both of which decline with age, compromising mobility and independence [4]. In this context, power or high-velocity resistance training (HVRT), with moderate to high loads, enhances fall-related, gait, and activities of daily living functions—such as stair climbing performance or reactive balance—mainly through improvements in knee flexion-extension power [5,6]. Maximum strength training (Max) remains the reference and traditional strategy to improve muscle strength and hypertrophy, counteracting sarcopenia through combined neural and structural gains, and enhancing bone loading and metabolic health [7,8,9]. In turn, accentuated eccentric (Aecc) training elicits high mechanical tension with reduced internal load and metabolic cost, making it suitable for individuals with comorbidities or low exercise tolerance, while promoting robust strength, structural, and osteogenic adaptations, improving deceleration control, and connective tissue resilience [10,11,12,13].

Bone mineral density (BMD) and T-score are commonly used indicators of skeletal status and fracture risk, whereas bone turnover markers (BTMs)—type I procollagen N-terminal propeptide (P1NP; formation) and beta C-terminal cross-linked telopeptide of type I collagen (β-CTX; resorption)—provide early insight into bone metabolism and responsiveness to training [14]. HVRT elicits modest yet significant BMD gains in the spine, femoral neck, and hip, with potential dose-response effects (≥2 sessions/week) [6]. Moderate-to-high load resistance training helps preserve BMD and may improve bone microarchitecture [8,12]. Therefore, integrating BMD and BTMs provides complementary insight into both early and structural skeletal adaptations.

Evaluating the neuromuscular performance at different contraction velocities provides valuable insight into the age-related decline in strength and power that underlies functional limitations in older adults. At 60°/s, mechanical tension and time under tension explain the hierarchy of Max ≥ Aecc ≥ HVRT, consistent with strength-oriented adaptations and the comparable efficacy of one versus two Aecc sessions per week [9,10]. At 180°/s, velocity specificity favors HVRT ≈ Aecc ≥ Max; HVRT enhances the rate of force development (RFD), whereas Aecc and Max improve high-velocity motor coordination [15]. Assessing both velocities reflects daily functional demands—such as standing up, gait acceleration, or balance recovery—closely associated with fall and fracture risk [4,5]. These neuromuscular responses are primarily driven by neural mechanisms; as noted by Alix-Fages et al. [16], motor unit recruitment and synchronization play key roles in strength regulation, supporting the rationale for using resistance training modalities with distinct neural demands.

These mechanical and neural adaptations are modulated by systemic mediators. Reducing inflammaging and oxidative stress through well-dosed RT may enhance the osteomuscular environment and remodeling processes [17,18,19]. Although not bone-specific, brain-derived neurotrophic factor (BDNF) reflects neuromuscular plasticity and exerkine activity associated with strength and tissue remodeling [20]. RT has been shown to increase resting BDNF levels in older adults, reinforcing its role in neural adaptation [21]. Thus, BDNF may be analyzed as a potential link between reduced inflammaging and muscle-driven osteogenesis. Preclinical studies support BDNF/TrkB-mediated bone crosstalk—promoting osteoblastogenesis and reducing bone loss—although human findings remain context-dependent and sometimes inconclusive (e.g., increases in RANKL and bone resorption) [22,23,24]. Considering this integrative framework, BDNF could be included as a secondary outcome to explore its covariation with interleukin-6 and tumor necrosis factor alpha (IL-6/TNF-α), F2-isoprostanes and glutathione peroxidase (GPx), and isokinetic torque (60°/s and 180°/s). This approach may help clarify the indirect contribution of BDNF to bone health through immune-redox and neuromuscular mechanisms.

To our knowledge, this randomized controlled trial is the first to compare three EB-RT modalities (HVRT, Aecc, and Max) in sedentary older adults within a single randomized cohort. Compared with prior EB-RT trials that typically tested ≤2 modalities with few outcomes, this study concurrently evaluates (i) bone health indicators (P1NP, β-CTX, BMD, and T-score), (ii) neuromuscular function (isokinetic torque at 60°/s and 180°/s), and (iii) systemic mediators (IL-6, TNF-α, F2-isoprostanes, GPx, and BDNF), enabling modality-specific, multidomain comparisons to inform individualized prescriptions in healthy aging. This study aimed to compare these EB-RT modalities across bone-muscle domains and systemic biomarkers, hypothesizing that: (i) HVRT would enhance power and osteogenic signaling; (ii) Aecc would improve maximal isokinetic strength at lower velocities; (iii) Max would elicit stable anti-inflammatory and antioxidant effects alongside strength gains comparable to the other training groups.

## 2. Materials and Methods

### 2.1. Study Design

This 16-week, four-arm randomized controlled trial included: HVRT (high-velocity resistance training; *n* = 21), Aecc (accentuated eccentric resistance training; *n* = 13), Max (maximum resistance training; *n =* 10), and a non-exercising control group (*n* = 17). The study was conducted in accordance with the Declaration of Helsinki, approved by the Human Research Ethics Committee of the University of Valencia (IRB No. 1861154; 5 May 2022), and registered at ClinicalTrials.gov (NCT06620666) on 24 January 2024.

### 2.2. Participants

The inclusion criteria were the following: (i) sedentary adults aged >60 years; (ii) preserved autonomy (i.e., ability to walk 100 m and climb 10 stairs unaided); (iii) medical clearance for resistance exercise; (iv) no antioxidant use in the previous 6 weeks; (v) no history of tobacco or alcohol abuse. Exclusion criteria included the following: (i) major visual or auditory deficits; (ii) life expectancy <1 year; (iii) ≥10% body weight change in the past 12 months; (iv) use of confounding medications (e.g., hormone replacement therapy, calcitonin, glucocorticoids, allopurinol); (v) cognitive impairment (Mini-Mental State Examination score < 23); (vi) recent participation in other clinical trials (<6 months).

Participants were recruited via institutional mailing lists [Nau Gran program (University of Valencia, Spain) and the Senior University of the Polytechnic University of Valencia, Spain] and snowball sampling. All participants provided written-informed consent and could withdraw at any time.

### 2.3. Randomization and Blinding

Participants were randomly allocated (1:1:1:1) to groups via computer-generated simple randomization with no stratification or blocking. An independent investigator (not involved in recruitment, intervention, or analysis) generated and held the sequence. Concealment was ensured via centralized allocation: sequential codes were released only after eligibility confirmation and baseline completion, and recruiters had no access to the sequence or keys. Participants were aware of their training modality due to the nature of the intervention, but remained blinded to other group assignments. Outcome evaluators and statisticians were also blinded to group allocation. Thus, the study qualifies as a randomized, double-blind controlled trial.

### 2.4. Testing Procedures

#### 2.4.1. General Testing Protocol

All evaluations were conducted at the University of Valencia (Spain). Participants fasted for 8 h before blood collection and refrained from non-steroidal anti-inflammatory drugs (NSAIDs) for 7 days before both blood sampling and performance tests, and from caffeine and strenuous activity for 24 h. Baseline assessments were completed before the familiarization phase; post-intervention measurements occurred 72 h after training completion. Primary outcomes comprised bone parameters [BMD, T-score (femoral neck and lumbar spine), P1NP, β-CTX] and isokinetic strength (knee and elbow flexo-extension at 60/180°/s). Secondary outcomes included systemic biomarkers of inflammation, oxidative stress, and neuroplasticity. Assessments were scheduled at consistent time points (±2 h) under standardized environmental conditions. Each evaluation phase consisted of two sessions: the first (08:30–12:00) involved blood collection, and the second, conducted 72 h later, included anthropometry, bone densitometry, and isokinetic testing.

#### 2.4.2. Bone Health

BMD and T-scores at the femoral neck and lumbar spine (L2–L4), as well as total hip BMD, were assessed using fan-beam dual-energy X-ray absorptiometry (DXA) (QDRÒ Discovery Wi, Hologic Inc., Marlborough, MA, USA) with APEX software (v12.4, Hologic Inc., Marlborough, MA, USA). DXA is the gold standard for bone mass evaluation, offering validated protocols and T-score-based thresholds [25]. Axial sites were selected for their clinical relevance and sensitivity to age- and training-related changes, particularly in older adults [26].

Venous blood samples were used to assess BTMs: P1NP and β-CTX. These biochemical indices reflect ongoing bone remodeling and complement DXA-derived outcomes in both clinical and research settings [14].

#### 2.4.3. Neuromuscular Strength of Knee and Elbow

Isokinetic concentric strength of knee and elbow flexion-extension was assessed at 60°/s and 180°/s using a Biodex System 3.2 dynamometer (Biodex Medical, Shirley, NY, USA). After a standardized warm-up and familiarization (explanation on the function of the dynamometer and procedures for the test, mobility for the target joints, 2 × 5 repetitions with elastic bands for progressive overload, and 2 × 5 repetitions at maximum speed and minimum load on the isokinetic dynamometer), participants performed five maximal voluntary contractions with the dominant limb, with 2 min rest intervals between efforts [27]. Range of motion was set at 5–90° for the elbow and 0–90° for the knee. Standardized positioning and continuous verbal encouragements ensured measurement consistency.

#### 2.4.4. Collection and Processing of Blood Samples

Morning fasting venous samples were collected to reduce pre-analytical variability. Samples were centrifuged (1500–3000 rpm, 10–15 min, 4 °C), and serum aliquots were stored at −60 to −80 °C until analysis. Biomarkers were analyzed using commercially available ELISA kits (β-CTX: EK716118; P1NP: EK714439; BDNF: EIA5968; F2-isoprostanes: EIA3080; GPx: EK241437; IL-6: IL E3200; TNF-α: IL E3100; DRG Instruments, Marburg, Germany; and AFG Scientific, Northbrook, IL, USA). Concentrations were determined via four-parameter logistic regression and validated using GraphPad Prism 10 software (GraphPad Software, Boston, MA, USA). Assay coefficients of variation were <10%, and procedures adhered to Clinical and Laboratory Standards Institute (CLSI) guidelines.

#### 2.4.5. Anthropometric Measurements

Height (±0.1 cm) was measured using a SECA 217 stadiometer (Seca GmbH & Co., Hamburg, Germany). Body fat percentage (%) and body mass (kg) were evaluated via multifrequency bioelectrical impedance (Tanita^®^ MC 780-P MA, Tokyo, Japan). Participants wore light clothing and no metal items to ensure accuracy. Body mass index (BMI) was calculated as body mass (kg)/height^2^ (m^2^).

#### 2.4.6. Dietary and Physical Activity Control (Potential Confounder Variables)

Nutritional intake was assessed pre- and post-intervention via 3-day food logs (2 weekdays + 1 weekend day) using the validated MyFitnessPal application [28]. A certified nutritionist oversaw the process, providing individual guidance to ensure adherence and accuracy. Habitual physical activity (excluding intervention-related efforts) was evaluated using the International Physical Activity Questionnaire (IPAQ) [29].

#### 2.4.7. Compliance and Safety

**Figure 1:**
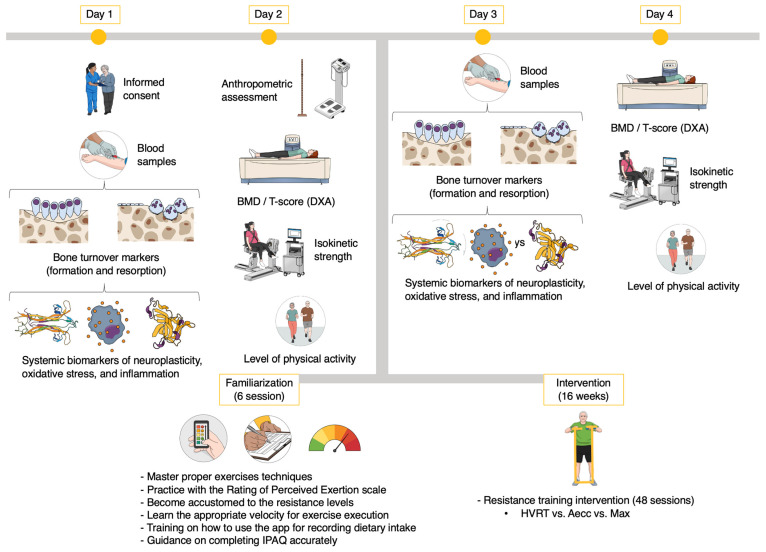
Study procedures. BMD, bone mineral density; DXA, fan-beam dual-energy X-ray absorptiometry; IPAQ, International Physical Activity Questionnaire; HVRT, high-velocity resistance training; Aecc, accentuated eccentric resistance training; Max, maximum resistance training.

Compliance was assessed via session attendance rate and self-reported supplementation adherence using standardized logs. Completion was defined as retention through post-intervention assessments, irrespective of session attendance. All intervention-related adverse events were documented. Figure 1 illustrates the study protocol.

### 2.5. Training Procedures

#### 2.5.1. Familiarization

Experimental groups completed six familiarization sessions to standardize exercise technique, movement tempo, and breathing coordination; calibrate resistance for elastic bands (EBs); and familiarize participants with rating of perceived exertion (RPE) using the OMNI-RES EB scale [30].

#### 2.5.2. Training Programs

All sessions were held at the University of Valencia (Spain). Over 16 weeks, participants completed 48 sessions (3/week, non-consecutive days, 48 h rest), each lasting ~60 min and following the American College of Sports Medicine (ACSM) guidelines [31]: 5–10 min warm-up (exercises for mobility, neuromuscular and cardiorespiratory activation with bodyweight, and range of movement), 40–50 min main training, and 5–10 min cooldown (dynamic slow stretches and mobility exercises, breathing and relaxation techniques). Sessions consisted of four sets of six or eight submaximal repetitions (depending on the training protocol) of multi-joint exercises for whole-body conditioning, alternating between upper- and lower-limb tasks in a fixed order and using CLX looped EB (TheraBand, The Hygenic Corporation, Akron, OH, USA). Training intensity was adjusted both objectively (by grip position, band color, and numbering; maximum elongation 300%; following published force-elongation calibrations [32,33]) and subjectively via RPE at the end of the first repetition of each set (RPE-1) [34,35]. A metronome standardized cadence. Active recovery (2 min between sets, 90 s between exercises) included low-intensity coordination and cognitive drills to enhance participants’ motivation and ensure adherence to the training program. All sessions followed safety standards for older adults [36] and were supervised by qualified sports scientists. RT protocols were progressive and individualized, with standardized execution parameters matched across groups in sets, reps, intensity, and intent.

Importantly, the exercise selection sought to replicate real-world, feasible training practices for older adults. In community contexts, elastic band exercises are typically performed self-anchored to the body whenever resistance levels are moderate—as implemented in the HVRT protocol. However, for higher-load conditions such as those required in Aecc and Max, self-anchoring is neither safe nor technically viable; therefore, anchored elastic band configurations were used to ensure ecological validity, safety, and reproducibility.

**Figure 2:**
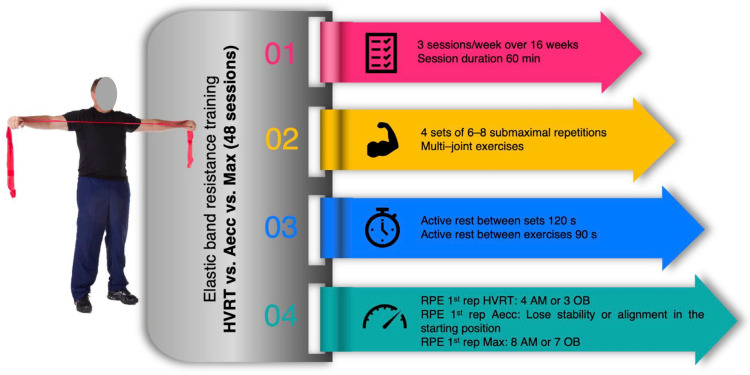
Experimental protocols for training intervention. HVRT, high-velocity resistance training; Aecc, accentuated eccentric resistance training; Max, maximum strength resistance training; RPE, rating of perceived exertion; AM, active muscles; OB, overall body.

Figure 2 summarizes the experimental protocols of the training intervention.

A. High-velocity elastic band resistance training program (HVRT)

Participants performed the following sequence: standing shoulder press, squat, upright row, lunges, and push press. The initial target RPE-1 was set at 4 for the active muscles or 3 for the overall body, corresponding to approximately 70% of the estimated one repetition maximum (1RM) [35]. Sets were also terminated when the RPE for the active muscles or the overall body reached values of 6 and 5, respectively, even if the initially prescribed eight submaximal repetitions were not completed [35]. EBs were marked and numbered symmetrically and in decreasing order every three centimeters from their center to ensure the reproducibility of the RPE-1 and symmetry in the grip [31]. Execution was metronome-paced: maximal intentional velocity concentric phase, 1-second isometric hold, and 2-second controlled eccentric, ensuring technical precision and safety.

B. Accentuated eccentric elastic band resistance training program (Aecc)

The main session consisted of four multi-joint exercises (lunges, standing chest presses, hip hinges, and horizontal rows) performed for six submaximal repetitions per set. EBs were anchored to a wall-mounted support and performed with both hands. Exercises began near the wall with an unloaded final concentric position, and participants moved backward or forward to increase band tension while maintaining proper joint alignment through an isometric posture. The point at which posture could no longer be maintained indicated the optimal resistance. Once optimal resistance was identified, a floor marker ensured consistency across sets. From this position, the eccentric phase was performed over 5 s. Then, the participant moved forward or backward, depending on the exercise, for 2 s toward the wall-mounted support to repeat the same procedure in the next repetition. This protocol ensured that participants exercised at loads slightly exceeding 100% of their estimated maximal concentric capacity [37] with RPE-1 values at the end of the eccentric phase typically reaching 7–8.

C. Maximal strength elastic band resistance training program (Max)

The exercise sequence, number of submaximal repetitions, and anchoring to the wall-mounted support were identical to those of the Aecc protocol. However, in this protocol, participants adjusted their position by moving forward or backward to modify band tension until reaching an RPE-1 of 8 for the active muscles and 7 for the overall body at the end of the concentric phase [34], corresponding to approximately 80–85% 1RM [38,39,40]. A floor marker was also placed to ensure consistent resistance across sets. Both concentric and eccentric phases were performed over two seconds.

D. Control group

The control group underwent only baseline and post-intervention assessments.

### 2.6. Statistical Analysis

In accordance with CONSORT guidelines [41], an intention-to-treat analysis was performed, carrying forward baseline values for participants who withdrew [42]. Data are reported as mean ± standard deviation (SD). Normality was assessed via the Shapiro-Wilk test. For normally distributed variables, a two-way repeated-measures ANOVA (time × group) was used. Post hoc comparisons were conducted using the Least Significant Difference (LSD) test. Additionally, a two-way repeated-measures ANCOVA was applied to adjust for baseline differences, using the baseline as covariates.

For non-normally distributed variables, the Kruskal-Wallis test assessed between-group differences. When baseline differences emerged, Quade’s rank ANCOVA was applied with the baseline as covariates [43]. Effect sizes (ES) were calculated as partial eta squared (ηp^2^) and interpreted as small (<0.06), moderate (0.06–0.14), or large (>0.14). Time effects were examined using the Friedman test, with effect sizes estimated via Kendall’s W and interpreted as ηp^2^. Post hoc tests included Wilcoxon signed-rank (within-group) and Mann-Whitney U (between-group) comparisons.

ES within groups were computed using Hedge’s *g,* while ES between groups were computed using the Morris *d_ppc_*_2_ approach for pre-post between-group designs, which standardizes gain scores using the pooled pre-test SD [44]. This method mitigates post-test bias and ensures stable estimates despite baseline imbalances. Simulations support its robustness and equivalence to other repeated-measures methods in moderately sized samples [44]. Cohen’s thresholds were used to classify ES as trivial (<0.20), small (0.20–0.49), moderate (0.50–0.79), and large (>0.80) [45]. Percentage changes (%Δ), and *d_ppc2_* and *g* values are reported in the “Results” tables.

Spearman’s rank-order correlations (*ρ*) were used to examine associations between pre-post changes in dependent variables. Correlation strength was classified according to standard thresholds [46], from negligible (0.00–0.10) to very strong (0.90–1.00). All analyses were conducted in SPSS software (version 28.0.1.1; IBM Corp., Armonk, NY, USA), with statistical significance set at *p* < 0.05.

### 2.7. Sample Size Determination

An a priori power analysis (G*Power 3.1.9.3) indicated that 56 participants were required to detect a moderate effect (*f* = 0.25) in a 4-group repeated-measures ANOVA, with α = 0.05 and 85% power. To offset a projected 15% attrition, the target sample size was increased to 64.

## 3. Results

### 3.1. Participant Flow and Baseline Characteristics

Of the individuals initially screened, a subset of 64 met the eligibility criteria, and 61 commenced the intervention. A total of 61 participants (33 women and 28 men) completed the 16-week program (for baseline characteristics, see Table 1).

**Figure 3:**
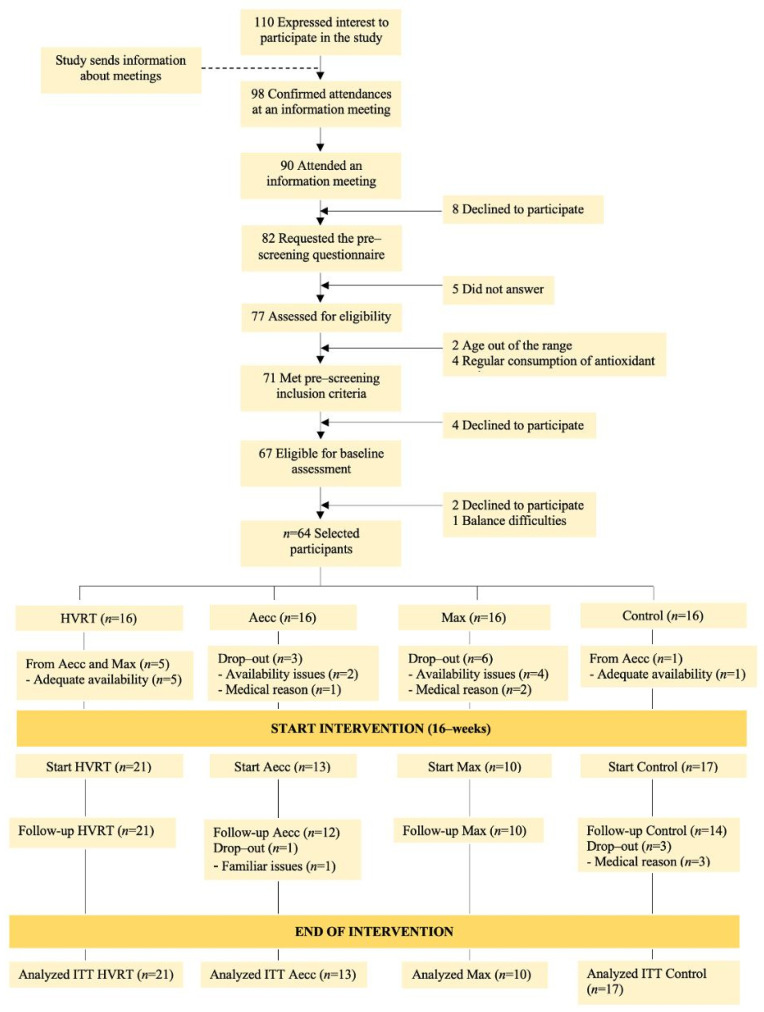
Participant flow throughout the study.

Although 16 participants were initially randomized to each group, the assigned training schedule—particularly in Aecc and Max—led to unequal sample sizes. Notably, several participants from the Aecc (*n* = 2) and Max group (*n* = 4) switched groups to continue participating in the study, specifically to the HVRT (*n* = 5) and the control group (*n* = 1). Please refer to Figure 3 for the participant flow diagram. Despite these modifications, randomization integrity was maintained, as no significant baseline differences were observed across demographic, physical activity, or dietary variables (all *p* > 0.05). The statistical methods employed (e.g., ANCOVA/Quade’s adjusted for baseline covariates) are robust to group size discrepancies and interindividual variability. Additionally, the intention-to-treat approach and post hoc power analysis based on the primary outcome (e.g., β-CTX) showed an effect size of *f* = 0.29 for the time × group interaction; α = 0.05; power ≥ 90% (1–β > 0.90). These results indicate that the total sample was sufficient to detect clinically relevant effects and minimize Type II error, supporting the robustness of the study design.

### 3.2. Program Feasibility and Safety: Compliance, and Adverse Events

Exercise program compliance averaged around 87%, with adherence rates exceeding 90% across all itervention groups (HVRT: 100%, Aecc: 87.50%, Max: 89.36%). High compliance, minimal attrition, and absence of adverse events support the program’s feasibility and safety in older adults.

### 3.3. Bone Health Outcomes

#### 3.3.1. Bone Turnover Biomarkers

Time effects were significant for both BTMs (β-CTX: F = 3.81, *p* = 0.048, ηp^2^ = 0.07; P1NP: χ^2^ = 11.95, *p* < 0.001, W= 0.20). Significant time × group interactions were found for β-CTX (F = 33.87, *p* < 0.001, ηp^2^ = 0.66) and P1NP (F = 15.46, *p* < 0.001, ηp^2^ = 0.25). All experimental groups improved with significant differences compared to the control group, specifically Aecc achieved the greatest improvements in both BTMs (β-CTX: Aecc ES: −1.82; P1NP: Aecc ES = 1.21). However, no statistically significant differences were detected among the experimental groups. Pairwise comparisons are in Table 2.

#### 3.3.2. BMD and T-Score of Femoral Neck, Total Spine, and Total Lumbar Spine

Time effects were significant for BMD of femoral neck (F = 4.13, *p* = 0.010, ηp^2^ = 0.18), BMD of total hip (F = 5.29, *p* = 0.025, ηp^2^ = 0.09), T-score of total hip (F = 6.21, *p* = 0.016, ηp^2^ = 0.10), and T-score of total lumbar spine (F = 9.03, *p* = 0.004, ηp^2^ = 0.14). No significant interaction of time effects was found for the T-score of the femoral neck (F = 2.17, *p* = 0.146), and BMD of the total lumbar spine (F = 0.05, *p* = 0.816).

A significant time × group interaction was found for the BMD of total hip (F = 3.17, *p* = 0.031, ηp^2^ = 0.14) and T-score of total lumbar spine (F = 4.17, *p* = 0.046, ηp^2^ = 0.07), with all experimental groups maintaining stable values and control declining. No significant differences emerged between groups for the BMD of femoral neck (F = 1.32, *p =* 0.255), T-score of femoral neck (F = 0.65, *p* = 0.589), T-score of total hip (F = 1.16, *p* = 0.333), and BMD of total lumbar spine (F = 0.67, *p* = 0.574). Pairwise comparisons appear in Table 3.

An exploratory analysis of BMD changes (mean change and proportion of participants that maintained or improved) at the femoral neck, total hip, and total lumbar spine, stratified by sex and intervention group, can be found in Supplementary Material, Figures S1–S6.

### 3.4. Isokinetic Strength Outcomes

Time effects were significant for all variables (all *p* < 0.001), confirming performance improvements (W: 0.32–0.53). Strength gains at 180°/s in the knee were most pronounced in Aecc, with a very large average ES (1.94). Strength gains at 180°/s in the elbow were most pronounced in Max, with a very large average ES (1.38). Regarding strength gains at 60°/s in the knee and elbow, the Max group showed better results with a very large average ES (knee: 1.17; elbow: 1.38). Significant time × group interactions were observed for all strength variables, with greater gains in experimental groups and declines or non-significant changes in control (knee flexion at 180°/s: F = 28.47, *p* < 0.001, ηp^2^ = 0.47; knee extension at 180°/s: F = 18.92, *p* < 0.001, ηp^2^ = 0.32; elbow flexion at 180°/s: F = 32.42, *p* < 0.001, ηp^2^ = 0.54; elbow extension at 180°/s: F = 37.56, *p* < 0.001, ηp^2^ = 0.62; knee flexion at 60°/s: F = 33.24, *p* < 0.001, ηp^2^ = 0.55; knee extension at 60°/s: F = 20.88, *p* < 0.001, ηp^2^ = 0.35; elbow flexion at 60°/s: F = 38.10, *p* < 0.001, ηp^2^ = 0.63; elbow extension at 60°/s: F = 40.73, *p* < 0.001, ηp^2^ = 0.68). See Table 4 for detailed comparisons.

### 3.5. Systemic Biomarkers of Oxidative Stress, Inflammation, and Neuroplasticity Outcomes

#### 3.5.1. Oxidative Stress

Time effects were significant for both F2-isoprostanes (χ^2^ = 4.27, *p* = 0.039, W = 0.07) and GPx (χ^2^ = 6.18, *p* = 0.028, W = 0.10). A significant time × group interaction was also observed (F2-isoprostanes: F = 21.47, *p* < 0.001, ηp^2^ = 0.36; GPx: F = 6.06, *p* = 0.001, ηp^2^ = 0.10) with F2-isoprostanes decreasing and GPx increasing in the intervention groups, while both worsened in controls. The Aecc group achieved the greatest improvements in F2-isoprostanes with a large ES (−0.94), and GPx with a moderate ES (0.40). However, no statistically significant differences were detected among the experimental groups. Pairwise comparisons are presented in Table 5.

#### 3.5.2. Inflammation

Time effects were significant for both IL-6 (χ^2^ = 6.69, *p* = 0.025, W = 0.11) and TNFα (F = 16.29, *p* < 0.001, ηp^2^ = 0.23). A significant time × group interaction was also observed for all inflammation biomarkers (IL-6: F = 21.04, *p* < 0.001, ηp^2^ = 0.35; TNFα: F = 23.15, *p* < 0.001, ηp^2^ = 0.29). All training groups significantly reduced both biomarkers, with Max showing the greatest improvements in IL-6 (large ES: −1.13), and TNF-α (moderate ES: −0.70). Additionally, the control group experienced an increase in both biomarkers. Pairwise comparisons are presented in Table 5.

#### 3.5.3. Neuroplasticity

A significant main effect of time was observed (BDNF: F = 3.03, *p* = 0.042, ηp^2^ = 0.05), reflecting overall improvements from baseline to the 16-week follow-up. A significant time × group interaction was found (BDNF: F = 9.32, *p* < 0.001, ηp^2^ = 0.35). All training groups improved BDNF levels, with the greatest increase in the HVRT group (*p* = 0.019; ES: 0.42), followed by Aecc (*p* = 0.020; ES: 0.25) and Max (*p* = 0.375; ES: 0.13). In contrast, BDNF significantly declined in the control group (*p* < 0.001; ES: −0.31). However, no statistically significant differences were detected among the experimental groups. Detailed pairwise comparisons are presented in Table 5.

### 3.6. Association Between Variables (Spearman ρ)

Spearman correlations on Δpre-post changes revealed consistent associations between bone health and neuromuscular strength. Strength gains, especially in knee and elbow extension/flexion, correlated positively with P1NP (e.g., knee flexion at 180°/s, *ρ =* 0.59, *p* < 0.001; knee extension at 60°/s, *ρ* = 0.55, *p* < 0.001) and negatively with β-CTX (e.g., knee extension at 60°/s, *ρ =* −0.65, *p* < 0.001; elbow extension at 180°/s, *ρ =* −0.62, *p* < 0.001). Similarly, changes in BMD and T-score (total hip and femoral neck) showed moderate positive correlations with strength (*ρ =* 0.27–0.39, *p* < 0.05), supporting a functional link between strength and bone remodeling.

Lower levels of F2-isoprostanes, IL-6, and TNF-α consistently correlated with greater strength (e.g., F2-isoprostanes vs. knee flexion at 60°/s, *ρ =* −0.69, *p* < 0.001; TNF-α vs. knee extension at 180°/s, *ρ =* −0.67, *p* = 0.001), as well as with higher P1NP and BMD/T-score (e.g., IL-6 with P1NP, *ρ =* −0.61, *p* < 0.001; TNF-α with P1NP, *ρ* = −0.53, *p* < 0.001; TNF– α with total hip BMD, *ρ =* −0.47, *p* < 0.001). Higher GPx levels were also positively associated with strength (e.g., elbow extension at 180°/s, *ρ =* 0.59, *p* < 0.001), with P1NP (*ρ* = 0.53, *p* < 0.001), and negatively with β-CTX (*ρ* = −0.60, *p* < 0.001). BDNF further correlated with several strength indices (*ρ* = 0.33–0.48, *p* < 0.001), with P1NP (*ρ* = 0.49, *p* < 0.001), and β-CTX (*ρ* = −0.54, *p* < 0.001).

**Figure 4:**
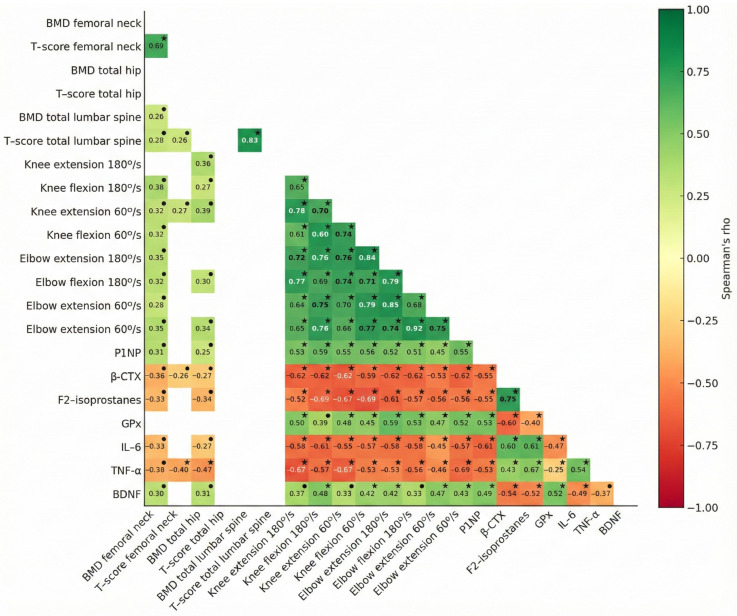
Association between pre-post intervention changes in dependent variables. The correlation magnitude is represented by color intensity, ranging from negative (red) to positive (green), with Spearman’s ρ displayed. Non-significant correlations are omitted. BMD, Bone mineral density; P1NP, Type 1 procollagen N-terminal propeptide; β-CTX, Beta C-terminal cross-linked telopeptide of type I collagen; GPx, Glutathione peroxidase; IL-6, Interleukin-6; TNF-α, Tumor necrosis factor-alpha; BDNF, Brain-derived neurotrophic factor. Significance markers: • *p* < 0.01; * *p* < 0.001.

These findings suggest that strength gains align with anabolic bone remodeling and that biomarkers of neuroplasticity, oxidative stress, and inflammation modulate these adaptive responses. See Figure 4 for more details.

## 4. Discussion

The relevance of the present study lies in being, to our knowledge, the first to compare the effects of different resistance training modalities—high-velocity, accentuated eccentric, and maximal—on bone, neuromuscular, redox-inflammatory, and neuroplastic adaptations in sedentary older adults. This approach provides novel insights into how specific mechanical and neural demands influence systemic health, allowing for a more precise and individualized prescription of elastic resistance exercise in this population. These findings may contribute to addressing several of the most prevalent age-related conditions that impair functional capacity and quality of life. The following sections discuss these results in relation to the study objectives and the specific variables analyzed. The integrated assessment of bone, neuromuscular, and systemic biomarkers provides a comprehensive understanding of how distinct mechanical and neural demands drive adaptations in aging.

To enhance external validity, EB-RT is implementable in home and community settings with clear instructions and basic dose control. Evidence from progressive EB-RT in older adults shows functional gains with low adverse-event rates outside laboratory settings [47]. Equipment can be standardized by mapping band color and % elongation to approximate load (N) via empirical calibration [32] and recent predictive equations [33]. Recording initial working length and marking grips improve session-to-session tension reproducibility, enabling consistent dosing in partially supervised or self-guided programs while preserving protocol fidelity.

### 4.1. Bone Remodeling Induced by HVRT, Aecc, and Max: A Comparative Analysis

In the present study, HVRT promoted a favorable osteoanabolic environment, evidenced by significant increases in total hip BMD and lumbar T-score, along with a 21% rise in P1NP and a 21% reduction in β-CTX. These effects likely reflect the high strain rate of HVRT, which activates osteogenic pathways via osteocyte-mediated mechanotransduction—such as sclerostin inhibition and Wnt/β-catenin signaling—favoring bone formation over resorption [48]. A training frequency of at least two sessions per week, as implemented in our protocol, appears critical to surpass the mechanical threshold necessary for DXA-detectable changes [49]. BTMs captured these early responses prior to densitometric consolidation, in line with previous HVRT trials [6]. While some studies report heterogeneous BTM responses in older adults [50], our results reveal a net osteoanabolic pattern, potentially due to the sedentary but otherwise healthy profile of our participants and the sustained, high-intensity regimen. Acute evidence also indicates that HVRT may modulate serum biomarkers before structural bone adaptations emerge, reinforcing the utility of BTMs as sensitive indicators of osteogenic efficacy [51].

Correlation analysis further supported this interpretation, revealing significant associations between changes in P1NP and both lumbar T-score (*ρ* = 0.42, *p* < 0.001) and total hip BMD (*ρ* = 0.37, *p* < 0.05), suggesting that increased bone formation coincides with early densitometric gains. Collectively, these findings position HVRT as a potent strategy to stimulate bone remodeling in older adults through both mechanical and cellular mechanisms.

Aecc appears effective in promoting an osteoanabolic response in sedentary older adults, as reflected in the 29% increase in P1NP and 25% decrease in β-CTX. Although changes in BMD were not statistically significant, favorable trends in hip and lumbar T-scores indicate a pro-osteogenic environment that could lead to structural gains with extended interventions. This response is likely driven by the high mechanical tension during the eccentric phase, which enhances bone deformation and activates osteocytic pathways, such as sclerostin inhibition and Wnt/β-catenin signaling [12]. Singh et al. [12] highlighted that this loading type maintains osteogenic efficacy despite lower metabolic strain, making it particularly appropriate for older adults with reduced exercise tolerance. Furthermore, the age-related preservation of eccentric force allows for effective loading stimuli, even in the context of sarcopenia or functional limitations [12]. Few studies have concurrently assessed the effects of Aecc on both BTMs and BMD, particularly in sedentary older adults. Recent reviews, including those by Kulkarni et al. [52] and Kim et al. [53], reported gains in strength, function, and balance following eccentric interventions, yet lacked BMD/BTM outcomes.

Correlation data support this rationale: significant inverse associations between P1NP and β-CTX (*ρ* = −0.45, *p* < 0.01), indicating a shift toward bone formation dominance. These support biochemical remodeling despite limited structural gains and validate BTMs as early markers of osteoanabolic response to eccentric training.

Maximal strength training induced a moderately favorable osteometabolic profile, evidenced by a 17% increase in P1NP and an 18% reduction in β-CTX. Despite non-significant densitometric changes, the concordance with BTMs suggests a bone-forming biological environment. These results align with evidence supporting the osteogenic potential of high-load training (≥80–85% 1RM) when applied progressively under controlled conditions [54]. Low-repetition maximal strength training generates sustained muscular tension, enhancing bone strain without requiring high movement velocities [55]. This modality promotes type II fiber recruitment and acute IGF-1 and osteocalcin release, both linked to osteogenic signaling [56], possibly explaining its moderate yet clinically meaningful osteoanabolic effects, distinct from those elicited by HVRT or Aecc protocols. Muollo et al. [51] showed that high-intensity training can modulate P1NP and CTX-1 in older adults with limited mobility, though without concurrent BMD assessment. Tøien et al. [54] further highlight that very heavy load training is safe and effective in older adults, promoting functional and potential skeletal benefits even without velocity or eccentric overload components. A significant correlation supports this interpretation: P1NP was associated with gains in knee extension strength (*ρ* = 0.48, *p* < 0.01), indicating that high tension promotes neuromuscular and bone remodeling.

These findings identify HVRT, Aecc, and Max as effective strategies to stimulate bone adaptation in sedentary older adults, each with distinct physiological and mechanical profiles: velocity (HVRT), tension with low metabolic cost (Aecc), or sustained high load (Max). Correlations between biochemical and structural markers highlight the multimodal benefits of these interventions and the importance of tailoring exercise prescriptions to optimize skeletal health in aging.

### 4.2. Resistance Training Modalities and Their Differential Impact on Isokinetic Function

In the knee joint, flexion gains were highest in Max (78%; *g* = 1.28) and Aecc (70%; *g* = 2.32), followed by HVRT (47%; *g* = 1.56). For extension, Aecc led with 69% (*g* = 1.56), while Max and HVRT yielded comparable improvements (~50%). In the elbow joint, flexion gains were notably larger: Aecc (123%; *g* = 2.40), HVRT (108%; *g* = 1.46), and Max (104%; *g* = 1.04); similar trends were observed for extension, with Max (69%), Aecc (64%), and HVRT (51%).

These adaptations likely reflect shared neuromuscular mechanisms; Aecc elicits high sustained tension with low metabolic cost, promoting peripheral (hypertrophy, tendon stiffness) and central [motor recruitment, rate of force development (RFD)] adaptations, particularly effective in smaller distal muscles such as the elbow flexors [10,57]. Maximal training (≥80% 1RM) enhances type II fiber recruitment, neuromuscular synchronization, and tendon stiffness, supporting force transmission even under high-velocity demands [7,58]. Additionally, maximal intent during concentric contractions fosters neural adaptations transferable to dynamic tasks [59]. HVRT preferentially recruits high-threshold motor units and preserves type II fibers, enhancing rapid force production critical for postural recovery and gait initiation [60,61].

Spearman correlations revealed a significant association between improvements in isokinetic strength and BTMs. Torque at 180°/s (flexion and extension) was positively correlated with changes in P1NP (*ρ* = 0.51–0.59, *p* < 0.001) and β-CTX (*ρ* = −0.62, *p* < 0.001), indicating a tight coupling between neuromuscular gains and bone turnover.

At 60°/s, all three training modalities improved isokinetic strength in both joints, albeit with smaller gains than at 180°/s—likely due to reduced explosive demands. In the knee, flexion improved most in Max (60%; g = 1.43), followed by Aecc (47%; g = 1.65) and HVRT (41%; g = 1.16), with significant between-group differences favoring Max and Aecc. Extension gains were more homogeneous: Max (43%; g = 0.91), Aecc (41%; g = 1.34), and HVRT (39%; g = 1.01). In the elbow, flexion gains ranked as follows: Max (66%; g = 1.32), Aecc (52%; g = 1.46), and HVRT (41%; g = 0.98). For extension, Max again led (49%; g = 1.28), followed by Aecc (45%; g = 1.24) and HVRT (38%; g = 0.85), with significant differences between Max and HVRT.

The use of lower velocities allows longer time under tension and heavier recruitment. These findings are consistent with Muollo et al. [51], who reported strength benefits in older adults using similar protocols. Aecc proved especially effective in the knee due to its ability to generate high tension with low metabolic cost, fostering neural adaptations even at moderate velocities [62]. Although HVRT yielded smaller gains, it remained effective under submaximal velocities, albeit with less mechanical specificity. Correlation analysis further supported this interpretation, indicating significant associations between changes in torque at 60°/s (flexion and extension) and P1NP (*ρ* = 0.45–0.56, *p* < 0.001), and β-CTX (*ρ* = −0.55–−0.65, *p* < 0.001).

These findings highlight the importance of velocity-specific neuromuscular adaptations in aging. Aecc enhanced RFD and HVRT improved the responsiveness at higher velocities, while Max training was more effective at lower velocities, promoting torque and functional gains through mechanical and endocrine pathways.

### 4.3. Redox Dose-Response: Which Strength Training Modality Provides Greater Cellular Protection?

The redox-inflammatory balance is closely linked to bone metabolism. Mechanistically, chronic oxidative stress promotes osteoclastogenesis and impairs osteoblast function, while antioxidant activity—such as that induced by GPx—activates Wnt/β-catenin signaling and enhances bone formation [63,64]. This is consistent with Zhou et al. [65], who reported a negative association between the systemic inflammatory response index (SIRI) and P1NP/β-CTX in osteoporotic patients, indicating that higher systemic inflammation is associated with suppressed bone turnover. However, this study demonstrates that a 16-week RT program, regardless of modality, induces parallel redox-inflammatory and osteometabolic adaptations in sedentary older adults.

The intervention improved the redox-inflammatory profile, with marked effects in Aecc and HVRT groups. F2-isoprostanes, a marker of oxidative stress, decreased by −29% in Aecc (*p* = 0.007: *g* = −0.94), Max (*p* = 0.017; *g* = −0.68), and −23% in HVRT (*p* = 0.002; *g* = −0.62), contrasting with a 36% increase in the control group (*p* < 0.001; *g* = 1.15). GPx, a key antioxidant enzyme, increased in HVRT (7%, *p* < 0.001) and Aecc (6%, *p* = 0.020), remained unchanged in Max (*p* = 0.721), and declined in control (−8%, *p* < 0.001).

IL-6 levels significantly decreased in all training groups—Aecc (−20%), HVRT (−14%), and Max (−13%)—with *p* < 0.01 and large ES (*g* ≈ −0.85 to −1.13), while the control group showed a 51% increase (*p* < 0.001; *g* = 1.63). TNF-α also declined in Aecc, Max (both −9%), and HVRT (−8%) (all *p <* 0.005), but remained unchanged in control. These changes align with evidence that RT reduces pro-inflammatory cytokines in older adults via IL–10, irisin, and activation of the AMPK-PGC1α axis [66,67].

In our study, groups with greater redox improvements (e.g., Aecc) also exhibited higher P1NP and lower β-CTX levels, reinforcing the redox-bone turnover link. This is supported by significant correlation between GPx and P1NP (*ρ* = 0.53, *p* < 0.001), and β-CTX (*ρ* = −0.60, *p* < 0.001), and IL-6/TNF-α with P1NP (*ρ* = −0.53–−0.61, *p* < 0.001).

From a physiological standpoint, these adaptations suggest that RT moderately activates pro-oxidant pathways, which, when kept below harmful thresholds, trigger hermetic responses in the antioxidant system [68]. HVRT, through rapid, high-velocity contractions, may enhance mitochondrial demand and GPx expression via transient oxidative stress [69]. In contrast, Aecc induces sustained tension and redox signaling through controlled muscle strain, promoting adaptation without triggering systemic inflammation [62].

The Max group showed significant reductions in oxidative stress and systemic inflammation, but GPx activity remained unchanged. This suggests that heavy-load training, which is characterized by low movement velocity and lower motor complexity compared with other methodologies, provides immunometabolic benefits, but the lack of velocity or motor complexity may limit antioxidant adaptation. Therefore, maximum load alone may not be sufficient to enhance redox-inflammatory responses. In contrast, stimuli with greater complexity or velocity (e.g., HVRT, Aecc) appear to elicit stronger and more sustained redox-inflammatory responses, likely via combined mechanical, mitochondrial, and neural stimuli enhancing molecular adaptations [21,70]. Thus, the findings indicate that training intensity alone does not guarantee antioxidant benefits, highlighting the need for velocity- or complexity-based components to induce those specific adaptations.

These results support the immunomodulatory potential of RT in sedentary older adults and indicate that stimulus characteristics (tension, velocity, complexity) shape the magnitude and durability of effects on oxidative stress and systemic inflammation, with downstream implications for bone remodeling and functional health.

### 4.4. BDNF in Older Adults: Adaptive Differences According to Resistance Training Modality

The HVRT protocol increased basal BDNF (10%; *p* = 0.019), reinforcing its neuroplastic potential in older adults. This distinct response, compared to the intermediate effect in Aecc and the null result in Max, may reflect the explosive and coordinative demands of HVRT. In our trial, BDNF is interpreted as a putative marker of neuroplastic potential rather than an indicator of cognitive health, as no primary neurocognitive outcomes were assessed. This wording aligns with evidence that exercise elevates circulating BDNF with context-dependent effects and notable heterogeneity across modalities, sex, and sampling matrices [71]. This modality enhances fast motor unit recruitment and neural synchronization, activating cortical and subcortical regions involved in BDNF regulation [72,73]. Emerging evidence suggests that contraction and activation velocity, and motor complexity, play a greater role in modulating BDNF than absolute load or training volume. For example, Tøien et al. [59] found that maximal intended velocity enhances motor activation regardless of load; Castaño et al. [74] reported that combining resistance exercise with a cognitive task increased BDNF despite similar loading, and Cefis et al. [75] emphasized the importance of neuronal activity and sensory signaling. Recent reviews also support the notion that moderate-to-high intensity strength and power training can modulate BDNF in older adults, though findings vary by contraction type, velocity, and timing of assessment [21,76].

Consistent with our findings, the moderate BDNF response with Aecc and the lack of change in the Max group contrast with the increase seen in neurometabolically demanding HVRT protocols. These findings may reflect the shared neuromuscular profile of both modalities: high mechanical tension with low motoneuronal discharge and metabolic cost. In Aecc, this yields a mechanically efficient yet neurometabolically modest stimulus [77]. Similarly, maximal strength training, despite high loads (≥80% 1RM), is executed slowly and with low motor complexity, limiting the cortical activation needed for sustained BDNF release [78,79]. Hence, the BDNF response to Aecc and Max may be attenuated compared to HVRT, which combines velocity, motor complexity, and coordination to enhance neuroplasticity. This aligns with evidence linking BDNF upregulation to neuroendocrine activation, muscle remodeling, and sustained synaptic demand [80], underscoring the need to consider both load and motor stimulus in training prescription for neurotrophic gains in older adults.

Correlational analyses showed significant associations between BDNF and bone remodeling markers, namely a positive correlation with P1NP (*ρ* = 0.58, *p* < 0.01) and a negative correlation with β-CTX (*ρ* = −0.49, *p* < 0.05). These findings support a link between neuroplasticity and osteogenesis. Although human data are limited, animal studies show that BDNF also regulates bone metabolism, promoting osteoblast proliferation and inhibiting osteoclast activity via TrkB- and Wnt/β-catenin-mediated pathways [22,81]. Its peripheral expression may be modulated by muscle-derived signals that activate neuroendocrine and myokine pathways involved in bone remodeling, such as IGF-1 and osteocalcin [82]. These results suggest that post-intervention increases in BDNF (e.g., HVRT, Aecc) may support osteoanabolic adaptations by promoting bone formation and reducing resorption.

These findings support the use of individualized RT in sedentary older adults, tailored to clinical profiles and therapeutic goals. HVRT involving ~70% 1RM and explosive concentric actions is particularly suitable for active individuals or those at low fall risk, aiming to enhance neuromuscular velocity and synaptic plasticity. Aecc training, based on slow (~5 s) eccentric contractions involving loads exceeding 100% of 1RM and performed without concentric loading, is especially appropriate for older adults with osteometabolic fragility or chronic inflammation, as it effectively stimulates bone remodeling and modulates redox-immune status with minimal metabolic demand. Max training, using high loads (~80–85% 1RM) and few slow repetitions, is effective in counteracting functional decline, improving strength without neuroendocrine or inflammatory stress.

In light of these considerations, it is now possible to adapt strength training protocols to the individual needs of older adults to improve training safety and efficacy, leading to a more time-efficient and effective approach.

Several methodological limitations should be considered, despite the clinical relevance of the findings. First, the small sample size in some groups (notable Max and Aecc) may have reduced statistical power to detect subtle effects, particularly in biomarkers and BMD. Although ANCOVA and Quade adjustments were applied, larger and more balanced samples are needed to enhance external validity. Second, the 16-week duration may be insufficient to elicit detectable structural bone changes in older adults with slower turnover. Longer follow-ups (>20 weeks) could clarify the durability of improvements and their clinical relevance (e.g., fracture risk). Third, although the training protocols were effective for functional and metabolic outcomes, they were not designed to optimize skeletal adaptations. The intervention was not explicitly designed to maximize osteogenesis, which typically requires tailored programming based on known osteogenic principles (e.g., localized loading, higher impact exercises, progressive overload targeting osteosensitive regions).

Future studies should examine the differential impact of these protocols on bone microarchitecture [e.g., via high-resolution peripheral quantitative computed tomography (HR-pQCT)] and include neuroimaging [functional magnetic resonance imaging (fMRI), electroencephalogram (EEG)] to characterize cortical responses. Additionally, longer duration studies (e.g., 6–12 months) should confirm whether the observed favorable osteoanabolic shift eventually translates into BMD gains. Investigating individual variability (e.g., responders vs. non-responders) and incorporating genetic/epigenetic markers could clarify mechanisms underlying neuroendocrine, redox, and inflammatory adaptations. Multicenter randomized trials with a priori stratification by sex and baseline BMD and pre-specified responder/non-responder analyses would enhance power and external validity. To mechanistically triangulate BTMs, biomarker panels should include osteocytic/Wnt and osteoclast regulators—sclerostin, DKK1, OPG/RANKL—plus IGF-1. Concerning maximizing skeletal adaptations, future studies should adopt bone-targeted protocols by adjusting key variables such as movement type, loading frequency, ground reaction forces, and stimulus duration to promote site-specific osteoanabolic responses, while prospectively monitoring dietary calcium and vitamin D as covariates.

## 5. Conclusions

This study shows that different RT modalities improve bone, neuromuscular, redox-inflammatory, and neuroplastic potential in sedentary older adults. Although no significant between-group differences were observed, modality-specific trends emerged based on percentage changes. Partially in line with our hypotheses, HVRT and Aecc were particularly effective in modulating bone turnover markers (P1NP, β-CTX), neuroplasticity (BDNF), and redox-inflammatory biomarkers (GPx, IL-6), while the Max group produced greater gains in strength and reductions in oxidative and inflammatory markers (F2-isoprostanes, TNF-α). These findings support the application of all three RT modalities according to individual clinical and functional profiles, emphasizing the importance of personalized exercise prescriptions to optimize musculoskeletal and systemic health in aging. Specifically, HVRT is recommended when prioritizing neuroplasticity and functional power outcomes, Aecc when seeking broad osteometabolic and redox benefits, and Max when emphasizing maximal strength development. These findings support the use of modality-specific EB-RT as an effective and individualized strategy to promote healthy aging.

## Figures and Tables

**Table 1:** Baseline characteristics of participants across study groups.

Variable	HVRT	Aecc	Max	Control
Age (years)	69.10 ± 5.01	69.50 ± 4.00	69.50 ± 4.70	69.55 ± 4.73
Weight (kg)	69.51 ± 12.85	69.50 ± 12.00	71.10 ± 11.10	70.41 ± 10.86
Height (cm)	162.70 ± 7.52	163.20 ± 7.20	165.90 ± 7.00	166.51 ± 5.64
Body mass index (kg/m^2^)	26.26 ± 5.43	26.80 ± 2.90	25.70 ± 3.40	25.40 ± 4.28
Body fat percentage (%)	36.41 ± 6.36	34.40 ± 5.60	33.80 ± 5.80	35.15 ± 8.20
Total calories (kcal)	1672.18 ± 332.75	1774.95 ± 376.59	1593.70 ± 253.95	1750.03 ± 269.61
Weekly physical activity (MET-min/week)	705.00 ± 184.75	730.00 ± 209.98	805.01 ± 220.01	715.00 ± 185.25

Note: HVRT: elastic band high-velocity resistance training; Aecc: elastic band accentuated eccentric resistance training; Max: elastic band maximum resistance training; Control: non-training group.

**Table 2:** Intervention effects on the bone turnover biomarkers.

Variable	Group	Mean (SD) (Pre-Test)	Mean (SD) (Post-Test)	△%	*p*-Value (Time)	ES (Time)	Comparison (Group)	*p*-Value (Group)	ES (Group)
β-CTX (pg/mL)	(1) HVRT	283.47 (53.62)	224.67 (54.45)	−20.74	<0.001 *	−1.09	1–4	<0.001	−3.30
							2–4	<0.001	−5.54
	(2) Aecc	252.51 (34.27)	188.63 (35.94)	−25.29	<0.001 *	−1.82	3–4	<0.001	−4.38
		
	(3) Max	264.33 (42.47)	216.70 (42.73)	−18.01	<0.001 *	−1.12			
		
	(4) Control	163.58 (15.55)	243.97 (26.33)	49.14	<0.001 *	3.72			
		
P1NP (µg/L)	(1) HVRT	3.05 (0.83)	3.69 (0.99)	20.98	<0.001 *	0.70	1–4	<0.001	1.84
							2–4	<0.001	2.35
	(2) Aecc	2.46 (0.62)	3.17 (0.55)	28.86	0.001 *	1.21	3–4	<0.001	2.17
		
	(3) Max	2.86 (0.46)	3.34 (0.43)	16.78	0.005 *	1.08			
		
	(4) Control	3.56 (0.58)	2.83 (0.81)	−20.51	<0.001 *	−1.04			
		

Note: Specifically, β-CTX was analyzed with parametric ANCOVA. P1NP were analyzed using Quade’s non-parametric ANCOVA. SD: standard deviation; △%: percentage of change; ES (time): effect size measured by Hedge’s *g*; ES (group): effect size measured by Morris *d_ppc2_;* β-CTX: beta C-terminal cross-linked telopeptide of type I collagen; P1NP: type I procollagen N-terminal propeptide; HVRT: elastic band high-velocity resistance training group; Aecc: elastic band accentuated eccentric resistance training group; Max: elastic band maximum resistance training group; Control: control group. Significant *p*-values for within-group comparisons are highlighted with *.

**Table 3:** Intervention effects on the BMD and T-score of the femoral neck, total hip, and total lumbar spine.

Variable	Group	Mean (SD) (Pre-Test)	Mean (SD) (Post-Test)	△%	*p*-Value (Time)	ES (Time)	Comparison (Group)	*p*-Value (Group)	ES (Group)
Femoral neck BMD (g/cm^2^)	(1) HVRT	0.66 (0.08)	0.67 (0.09)	1.51	0.006 *	0.12			
		
	(2) Aecc	0.73 (0.10)	0.73 (0.09)	0.00	0.743	0.00			
		
	(3) Max	0.64 (0.09)	0.65 (0.09)	1.56	0.215	0.11			
		
	(4) Control	0.71 (0.16)	0.70 (0.16)	−1.41	0.045 *	−0.06			
		
Femoral neck T-score (SD)	(1) HVRT	−1.83 (0.82)	−1.76 (0.69)	3.83	0.407	0.09			
		
	(2) Aecc	−1.34 (0.75)	−1.23 (0.70)	8.21	0.260	0.15			
		
	(3) Max	−1.92 (0.90)	−1.78 (0.85)	7.29	0.231	0.16			
		
	(4) Control	−1.44 (1.17)	−1.57 (1.16)	−9.03	0.692	−0.11			
		
Total hip BMD (g/cm^2^)	(1) HVRT	0.80 (0.12)	0.81 (0.13)	1.25	<0.001 *	0.08	1–4	0.003	0.13
		
	(2) Aecc	0.90 (0.13)	0.91 (0.12)	1.11	0.387	0.08			
		
	(3) Max	0.82 (0.11)	0.83 (0.12)	1.22	0.344	0.09			
		
	(4) Control	0.86 (0.19)	0.85 (0.19)	−1.16	0.472	−0.05			
		
Total hip T-score (SD)	(1) HVRT	−1.14 (0.85)	−1.02 (0.96)	10.53	0.008 *	0.13			
		
	(2) Aecc	−0.69 (0.84)	−0.61 (0.81)	11.54	0.130	0.10			
		
	(3) Max	−1.11 (0.95)	−1.05 (0.94)	5.04	0.344	0.06			
		
	(4) Control	−1.33 (0.70)	−1.40 (0.71)	−5.26	0.650	−0.10			
		
Total lumbar spine BMD (g/cm^2^)	(1) HVRT	0.85 (0.13)	0.86 (0.14)	1.18	0.077	0.07			
		
	(2) Aecc	0.98 (0.14)	0.98 (0.14)	0.00	0.798	0.00			
		
	(3) Max	0.88 (0.10)	0.88 (0.09)	0.00	0.632	0.00			
		
	(4) Control	0.92 (0.17)	0.92 (0.17)	0.00	0.809	0.00			
		
Total lumbar spine T–T-score (SD)	(1) HVRT	−1.90 (1.22)	−1.75 (1.32)	7.89	0.002 *	0.12	1–4	0.020	0.13
		
	(2) Aecc	−0.81 (1.15)	−0.73 (1.12)	9.88	0.181	0.07			
		
	(3) Max	−1.61 (0.92)	−1.50 (0.82)	6.83	0.110	0.13			
		
	(4) Control	−1.29 (1.41)	−1.31 (1.41)	−1.55	0.816	−0.01			
		

Note: Specifically, BMD and T-score of femoral neck, and T-score of total hip were analyzed with parametric ANOVA. BMD of total hip, and BMD and T-score of total lumbar spine were analyzed with parametric ANCOVA. SD: standard deviation; △%: percentage of change; ES (time): effect size measured by Hedge’s g; ES (group): effect size measured by Morris *d_ppc_*_2_; BMD: bone mineral density; HVRT: elastic band high-velocity resistance training group; Aecc: elastic band accentuated eccentric resistance training group; Max: elastic band maximum resistance training group; Control: control group. Significant p-values for within-group comparisons are highlighted with *.

**Table 4:** Intervention effects on the isokinetic strength of the knee and elbow.

Variable	Group	Mean (SD) (Pre-Test)	Mean (SD) (Post-Test)	△%	*p*-Value (Time)	ES (Time)	Comparison (Group)	*p*-Value (Group)	ES (Group)
Knee flexion at 60°/s (N·m)	(1) HVRT	47.28 (16.62)	66.61 (16.70)	40.88	<0.001 *	1.16	1–3	<0.001	−0.62
							1–4	<0.001	1.19
	(2) Aecc	49.10 (14.84)	72.33 (12.25)	47.31	<0.001 *	1.65	2–3	0.015	−0.42
							2–4	<0.001	1.44
	(3) Max	52.50 (24.32)	84.16 (17.54)	60.30	<0.001 *	1.43	3–4	<0.001	1.57
		
	(4) Control	50.68 (18.36)	48.84 (17.54)	−3.63	0.351	−0.12			
		
Knee extension at 60°/s (N·m)	(1) HVRT	81.90 (28.64)	114.03 (34.98)	39.23	<0.001 *	1.01	1–4	<0.001	0.99
							2–4	<0.001	1.11
	(2) Aecc	93.06 (27.37)	130.86 (27.41)	40.62	0.001 *	1.34	3–4	<0.001	0.99
		
	(3) Max	90.86 (41.16)	129.90 (42.51)	42.97	0.005 *	0.91			
		
	(4) Control	91.08 (39.97)	88.74 (39.06)	−2.57	0.338	−0.08			
		
Knee flexion at 180°/s (N·m)	(1) HVRT	40.02 (15.82)	58.68 (15.96)	46.63	<0.001 *	1.56	1–2	0.018	−0.46
							1–3	<0.001	−0.82
	(2) Aecc	37.76 (17.32)	64.24 (15.43)	70.13	0.001 *	2.32	1–4	<0.001	1.11
							2–4	<0.001	1.47
	(3) Max	43.30 (22.13)	77.12 (22.03)	78.11	0.005 *	1.28	3–4	<0.001	1.68
		
	(4) Control	47.14 (18.66)	46.36 (18.75)	−1.65	0.351	−0.05			
		
Knee extension at 180°/s (N·m)	(1) HVRT	49.50 (13.74)	74.19 (17.65)	49.88	<0.001 *	1.15	1–2	0.020	−0.66
							1–4	<0.001	1.33
	(2) Aecc	48.81 (12.43)	82.43 (16.31)	68.88	<0.001 *	1.56	2–4	<0.001	1.71
							3–4	<0.001	1.27
	(3) Max	58.84 (21.90)	88.08 (21.96)	49.69	<0.001 *	1.47			
		
	(4) Control	60.40 (23.46)	59.65 (23.74)	−1.24	0.770	−0.06			
		
Elbow flexion at 60°/s (N·m)	(1) HVRT	18.12 (9.70)	34.78 (11.72)	91.94	<0.001 *	1.52	1–3	0.005	−0.53
							1–4	<0.001	1.38
	(2) Aecc	23.15 (9.46)	43.09 (9.72)	86.13	0.001 *	2.01	2–4	<0.001	1.52
							3–4	<0.001	1.44
	(3) Max	21.91 (18.54)	45.67 (18.59)	108.44	0.005 *	1.23			
		
	(4) Control	23.74 (16.54)	21.87 (14.96)	−7.88	0.196	−0.14			
		
Elbow flexion at 60°/s (N·m)	(1) HVRT	33.50 (10.08)	53.34 (14.57)	50.25	<0.001 *	1.69	1–3	<0.001	−0.48
							1–4	<0.001	1.65
	(2) Aecc	42.42 (10.08)	62.20 (15.09)	46.63	0.002 *	1.49	2–3	0.019	−0.45
							2–4	<0.001	1.57
	(3) Max	39.97 (17.36)	66.08 (15.52)	65.32	0.005 *	1.52	3–4	<0.001	1.68
		
	(4) Control	40.66 (13.89)	40.41 (14.02)	−0.61	0.550	−0.04			
		
Elbow flexion at 180°/s (N·m)	(1) HVRT	16.20 (8.93)	33.77 (14.08)	108.46	<0.001 *	1.46	1–2	0.025	−0.73
							1–4	<0.001	1.59
	(2) Aecc	19.62 (8.50)	43.76 (10.84)	123.04	0.001 *	2.40	2–4	<0.001	2.07
							3–4	<0.001	1.37
	(3) Max	20.20 (18.10)	41.12 (20.27)	103.56	0.005 *	1.04			
		
	(4) Control	21.70 (13.52)	21.05 (12.53)	−2.99	0.393	−0.07			
		
Elbow flexion at 180°/s (N·m)	(1) HVRT	31.90 (11.57)	48.32 (12.32)	51.47	<0.001 *	1.35	1–2	0.032	−0.57
							1–3	0.005	−0.61
	(2) Aecc	36.00 (10.28)	58.92 (10.91)	63.67	0.001 *	2.09	1–4	<0.001	1.34
							2–4	<0.001	1.87
	(3) Max	35.57 (15.34)	59.99 (16.34)	68.65	0.005 *	1.48	3–4	<0.001	1.71
		
	(4) Control	39.18 (13.63)	38.47 (14.71)	−1.81	0.046 *	−0.07			
		

Note: All variables were analyzed with Quade’s non-parametric ANCOVA. SD: standard deviation; △%: percentage of change; ES (time): effect size measured by Hedge’s g; ES (group): effect size measured by Morris *d_ppc_*_2_; HVRT: elastic band high-velocity resistance training group; Aecc: elastic band accentuated eccentric resistance training group; Max: elastic band maximum resistance training group; Control: control group. Significant p-values for within-group comparisons are highlighted with *.

**Table 5:** Intervention effects on the oxidative stress, inflammatory, and neuroplasticity biomarkers.

Variable	Group	Mean (SD) (Pre-Test)	Mean (SD) (Post-Test)	△%	*p*-Value (Time)	ES (Time)	Comparison (Group)	*p*-Value (Group)	ES (Group)
F2-isoprostanes (pg/mL)	(1) HVRT	4260.93 (1812.88)	3268.01 (1336.53)	−23.30	0.002 *	−0.62	1–4	<0.001	−1.44
							2–4	<0.001	−1.80
	(2) Aecc	4496.52 (1646.88)	3185.02 (1027.28)	−29.16	0.007 *	−0.94	3–4	0.006	−1.71
		
	(3) Max	4693.97 (1947.32)	3313.40 (2037.99)	−29.41	0.017 *	−0.68			
		
	(4) Control	3788.55 (1283.65)	5155.85 (1036.05)	36.09	<0.001 *	1.15			
		
GPx (µIU/mL)	(1) HVRT	228.93 (54.50)	245.94 (56.39)	7.40	<0.001 *	0.31	1–4	<0.001	0.74
							2–4	0.009	0.88
	(2) Aecc	202.13 (28.18)	214.21 (30.67)	5.98	0.020 *	0.40			
		
	(3) Max	233.11 (48.06)	234.43 (49.94	0.57	0.721	0.03			
		
	(4) Control	236.87 (38.28)	217.79 (33.81)	−8.06	<0.001 *	−0.52			
		
IL-6 (pg/mL)	(1) HVRT	12.85 (1.78)	11.08 (1.60)	−13.77	<0.001 *	−1.03	1–3	0.050	0.05
							1–4	<0.001	−2.79
	(2) Aecc	13.44 (3.81)	10.77 (2.07)	−19.87	0.004 *	−0.85	2–3	0.015	−0.25
							2–4	<0.001	−2.24
	(3) Max	14.32 (1.94)	12.45 (1.24)	−13.05	0.007 *	−1.13	3–4	0.001	−2.52
		
	(4) Control	10.23 (3.08)	15.43 (3.18)	50.83	<0.001 *	1.63			
		
TNF-α (pg/mL)	(1) HVRT	3.42 (0.47)	3.13 (0.42)	−8.52	<0.001 *	−0.64	1–2	0.005	−0.04
							1–3	0.019	−0.12
	(2) Aecc	3.01 (0.46)	2.74 (0.35)	−8.95	<0.001 *	−0.64	1–4	0.012	−0.79
							2–4	<0.001	−0.74
	(3) Max	2.63 (0.29)	2.39 (0.37)	−9.14	0.004 *	−0.70	3–4	<0.001	−0.74
		
	(4) Control	2.95 (0.53)	3.06 (0.41)	3.89	0.174	0.24			
		
BDNF (pg/mL)	(1) HVRT	40,495.48 (9351.05)	44,543.64 (9907.90)	10.00	0.019 *	0.42	1–4	<0.001	0.73
							2–4	0.002	0.53
	(2) Aecc	39,222.33 (8741.69)	41,295.85 (8935.97)	5.29	0.020 *	0.25	3–4	0.010	0.41
		
	(3) Max	43,736.96 (9564.52)	44,827.51 (8376.60)	2.49	0.375	0.13			
		
	(4) Control	46,015.66 (9587.65)	43,052.07 (9103.29)	−6.44	<0.001 *	−0.31			
		

Note: Specifically, BDNF and TNF-α were analyzed with parametric ANCOVA. F2-isoprostanes, GPx, and IL-6 were analyzed with Quade’s non-parametric ANCOVA. SD: standard deviation; △%: percentage of change; ES (time): effect size measured by Hedge’s g; ES (group): effect size measured by Morris *d_ppc2_*; BDNF: brain-derived neurotrophic factor; GPx: glutathione peroxidase; IL-6: interleukin-6; TNF-α: tumor necrosis factor alfa; HVRT: elastic band high-velocity resistance training group; Aecc: elastic band accentuated eccentric resistance training group; Max: elastic band maximum resistance training group; Control: control group. Significant *p*-values for within-group comparisons are highlighted with *.

## Data Availability

All data generated or analyzed during this study are included in this published article. The databases are available upon reasonable request to the corresponding author.

## Supplementary Materials

The following supporting information can be downloaded at: https://www.mdpi.com/article/10.3390/healthcare13233129/s1, Figure S1: Exploratory pre-post analysis of femoral neck BMD in women across experimental intervention groups; Figure S2: Exploratory pre-post analysis of total hip BMD in women across experimental intervention groups; Figure S3: Exploratory pre-post analysis of total lumbar spine BMD in women across experimental intervention groups; Figure S4: Exploratory pre-post analysis of femoral neck BMD in men across experimental intervention groups; Figure S5: Exploratory pre-post analysis of total hip BMD in men across experimental intervention groups; Figure S6: Exploratory pre-post analysis of total lumbar spine BMD in men across experimental intervention groups. Ref. [83] is cited in the supplementary material file.

## Abbreviations

The following abbreviations are used in this manuscript:

EB-RT	Elastic band resistance training
HVRT	High-velocity resistance training
Aecc	Accentuated eccentric resistance training
Max	Maximal resistance training
OMNI-RES EB	OMNI-RES elastic band scale
1RM	One-repetition maximum
RPE–1	Rating of perceived exertion in the first repetition
BDNF	Brain-derived neurotrophic factor
GPx	Glutathione peroxidase
P1NP	Type I procollagen N-terminal propeptide
β-CTX	Beta C-terminal cross-linked telopeptide of type I collagen
RT	Resistance training
BMD	Bone mineral density
BTMs	Bone turnover markers
RFD	Rate of force development
TrkB	Tyrose receptor kinase B
RANKL	Receptor activator of nuclear factor kappa beta
IL-6	Interleukin–6
TNF-α	Tumor necrosis factor alpha
NSAIDs	Non-steroidal anti-inflammatory drugs
DXA	Fan-beam dual-energy X-ray absorptiometry
ELISA	Enzyme-linked immunosorbent assay
CLSI	Clinical and Laboratory Standards Institute
BMI	Body mass index
IPAQ	International Physical Activity Questionnaire
ACSM	American College of Sports Medicine
SD	Standard deviation
ANOVA	Analysis of variance
ANCOVA	Analysis of covariance
LSD	Least significant difference
ES	Effect size
AMPK	AMP-activated protein kinase
PGC–1α	Peroxisome proliferator-activated receptor gamma coactivator 1alpha
HR-pQCTD	High-resolution peripheral quantitative computed tomography
fMRI	Functional magnetic resonance imaging
EEG	Electroencephalogram
