# Supplementary material for: High-Velocity, Accentuated Eccentric, or Maximal Elastic Band Resistance Training? Effects of Resistance Training Modalities on Bone Health, Isokinetic Strength, and Systemic Biomarkers in Sedentary Older Adults: A Comparative Study
Source: Healthcare (Basel). 2025 Dec 1;13(23):3129. doi: 10.3390/healthcare13233129 (PMC12692264; doi:10.3390/healthcare13233129)
Supplement: Supplementary file 1 [file healthcare-13-03129-s001.zip › healthcare-3971905-supplementary.pdf]

Supplementary material

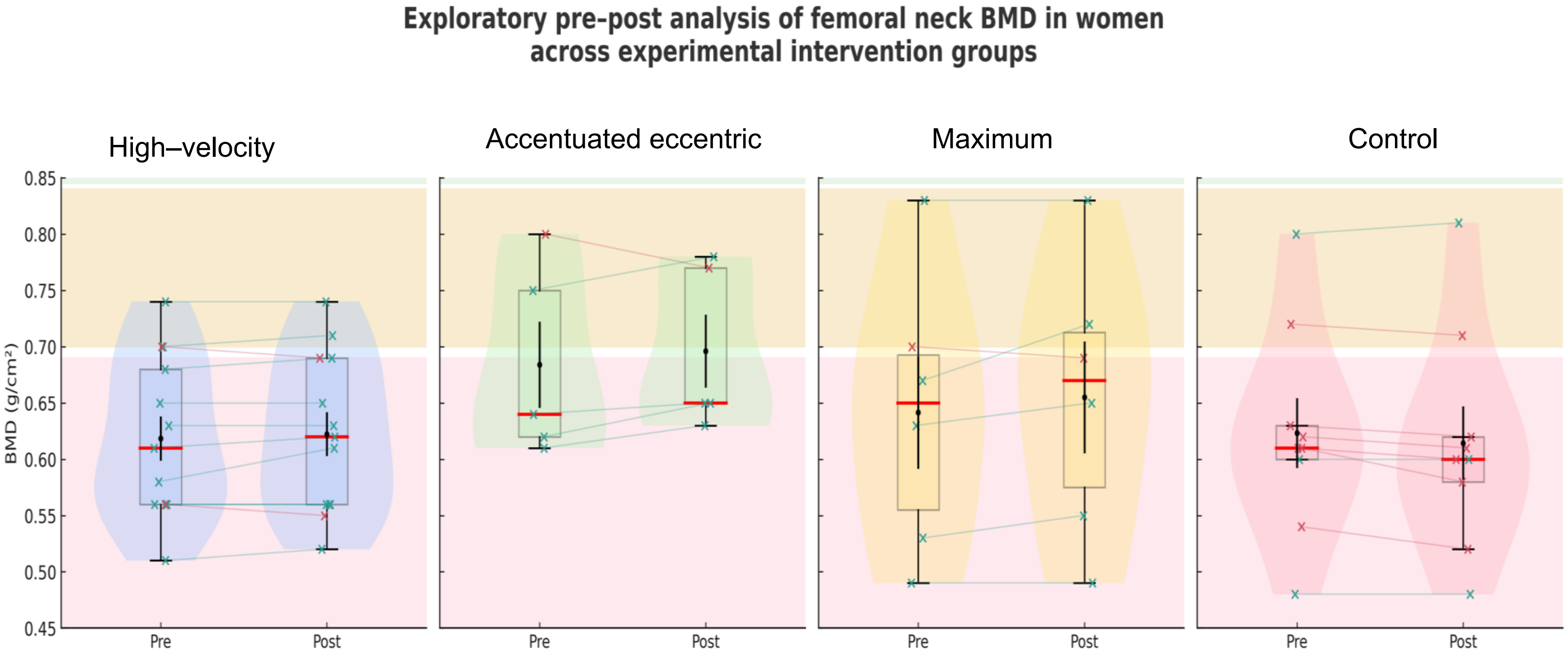

Figure S1. Exploratory pre–post analysis of femoral neck BMD in women across experimental intervention groups. Clinical bands: <0.69 (osteoporosis), 0.70–0.84 (osteopenia), ≥0.85 (normal) [83]. These visualizations are descriptive and do not imply statistical inference per se. BMD: bone mineral density.

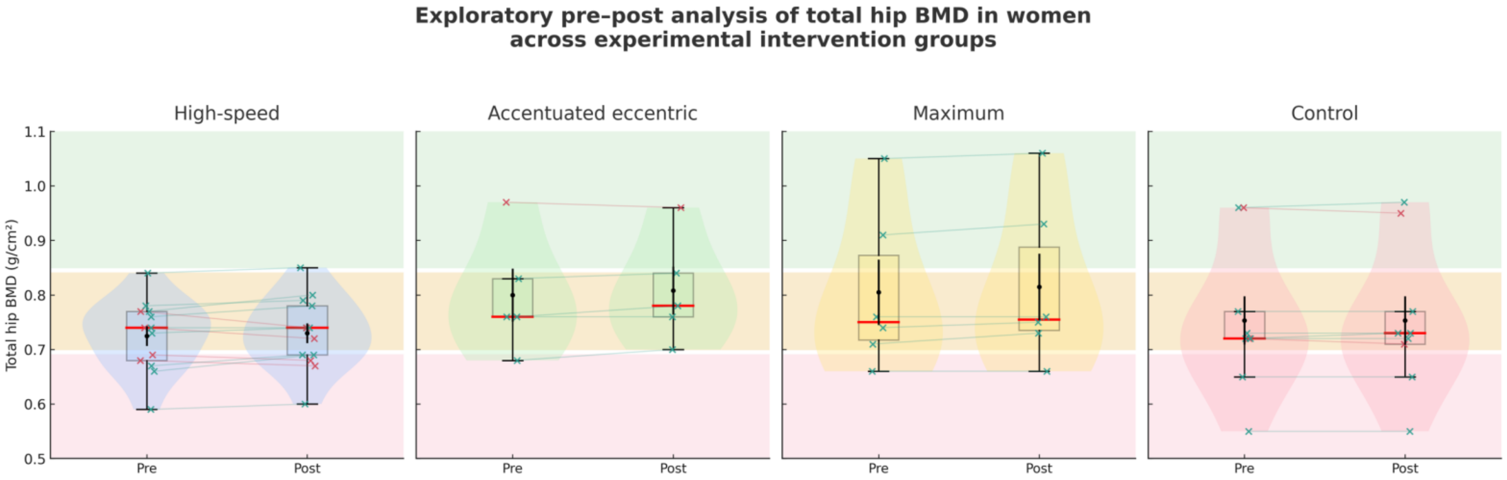

Figure S2. Exploratory pre–post analysis of total hip BMD in women across experimental intervention groups. Clinical bands: <0.69 (osteoporosis), 0.70–0.84 (osteopenia), ≥0.85 (normal) [83]. These visualizations are descriptive and do not imply statistical inference per se. BMD: bone mineral density.

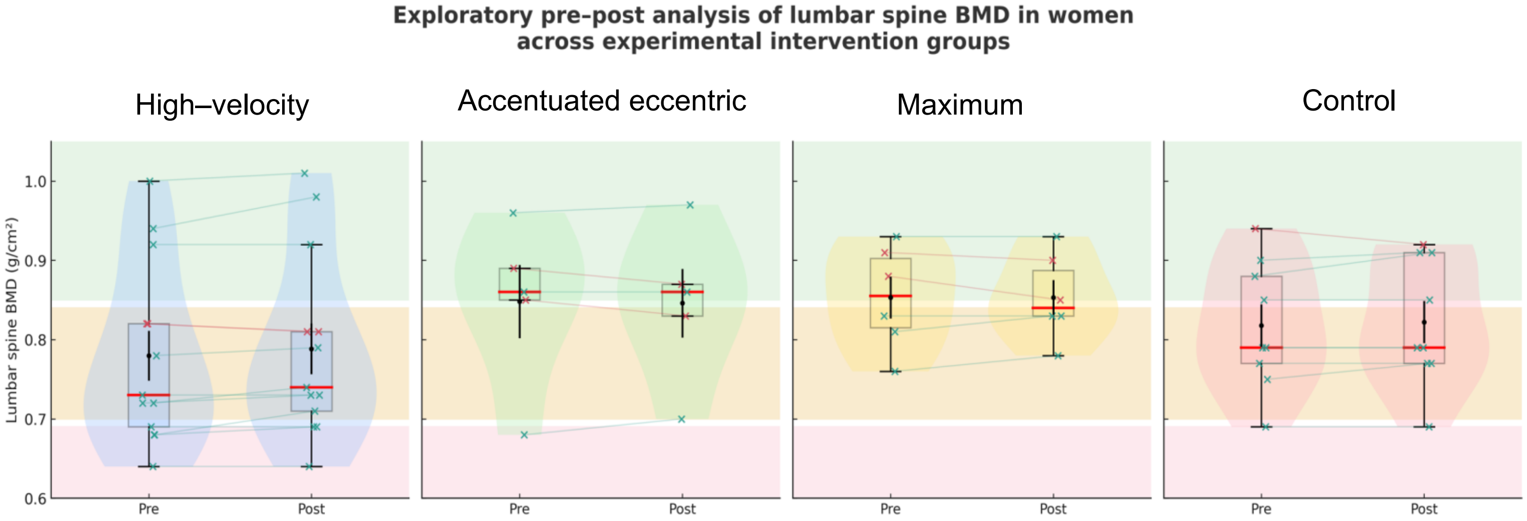

Figure S3. Exploratory pre–post analysis of total lumbar spine BMD in women across experimental intervention groups. Clinical bands: <0.69 (osteoporosis), 0.70–0.84 (osteopenia), ≥0.85 (normal) [83]. These visualizations are descriptive and do not imply statistical inference per se. BMD: bone mineral density.

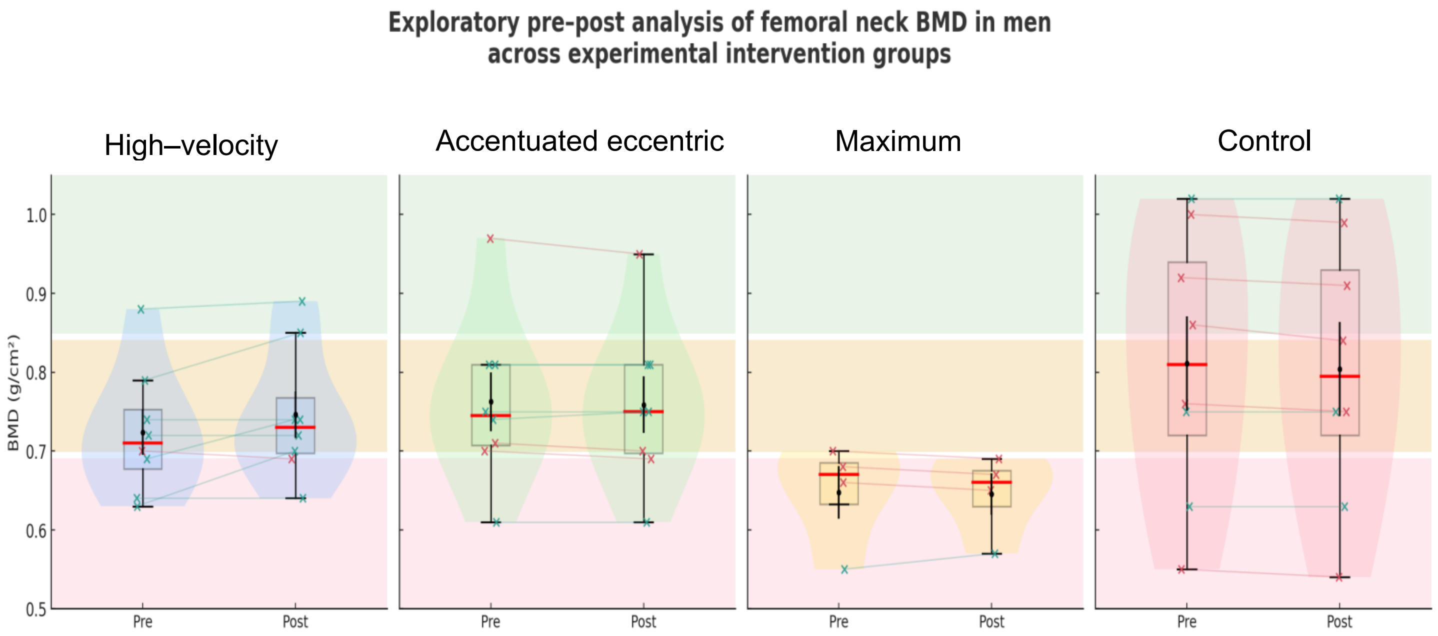

Figure S4. Exploratory pre–post analysis of femoral neck BMD in men across experimental intervention groups. Clinical bands: <0.69 (osteoporosis), 0.70–0.84 (osteopenia), ≥0.85 (normal) [83]. These visualizations are descriptive and do not imply statistical inference per se. BMD: bone mineral density.

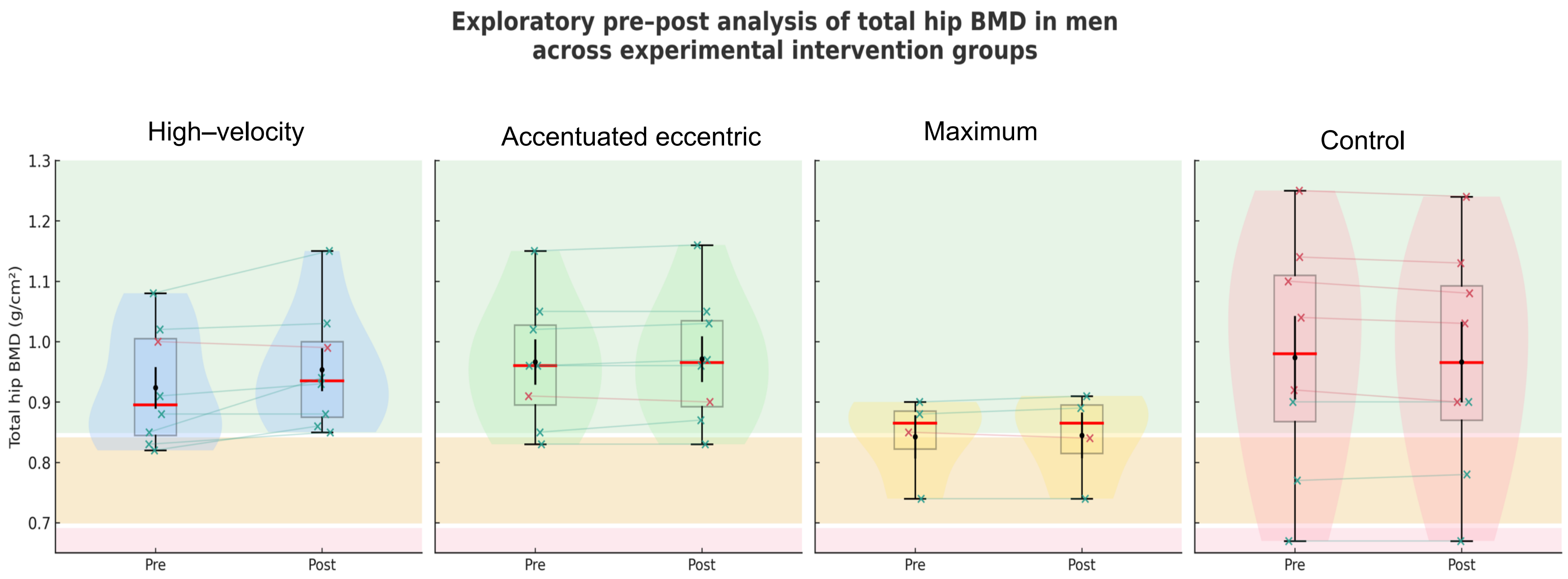

Figure S5. Exploratory pre–post analysis of total hip BMD in men across experimental intervention groups. Clinical bands: <0.69 (osteoporosis), 0.70–0.84 (osteopenia), ≥0.85 (normal) [83]. These visualizations are descriptive and do not imply statistical inference per se. BMD: bone mineral density.

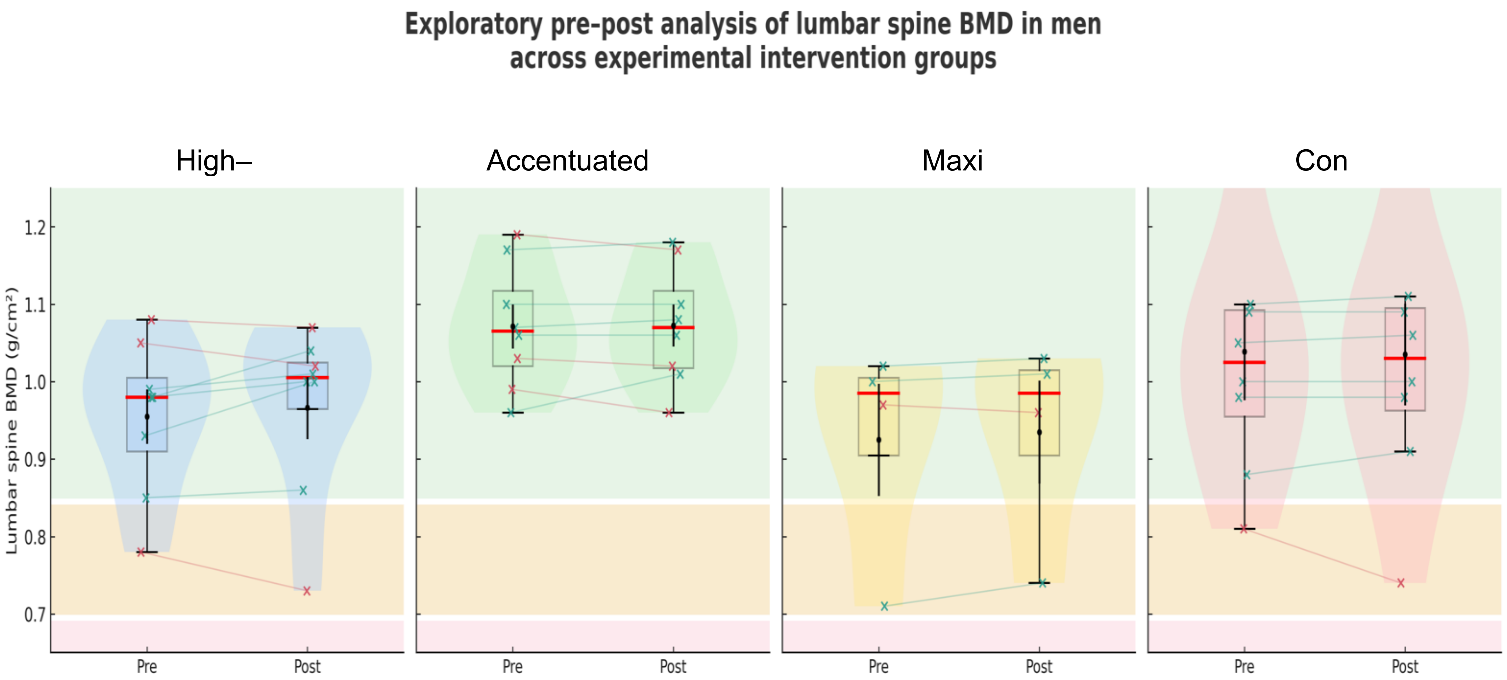

Figure S6. Exploratory pre–post analysis of total lumbar spine BMD in men across experimental intervention groups. Clinical bands: <0.69 (osteoporosis), 0.70–0.84 (osteopenia), ≥0.85 (normal) [83]. These visualizations are descriptive and do not imply statistical inference per se. BMD: bone mineral density.
